# Implication of Lactucopicrin in Autophagy, Cell Cycle Arrest and Oxidative Stress to Inhibit U87Mg Glioblastoma Cell Growth

**DOI:** 10.3390/molecules25245843

**Published:** 2020-12-10

**Authors:** Rossella Rotondo, Maria Antonietta Oliva, Sabrina Staffieri, Salvatore Castaldo, Felice Giangaspero, Antonietta Arcella

**Affiliations:** 1 Department of Molecular Medicine and Medical Biotechnologies, University of Naples Federico II, 80131 Naples, Italy; rossellaross1988@gmail.com; 2 I.R.C.C.S Neuromed, Via Atinense, 18, 86077 Pozzilli IS, Italy; mariaantonietta.oliva@neuromed.it (M.A.O.); sabrina.staffieri@neuromed.it (S.S.); castaldosal90@gmail.com (S.C.); felice.giangaspero@uniroma1.it (F.G.); 3 Department of Radiologic, Oncologic and Anatomo Pathological Sciences, University of Rome La Sapienza, 00185 Rome, Italy

**Keywords:** glioblastoma (GBM), lactucopicrin (LCTP), temozolomide (TMZ), autophagy, oxidative stress, NF-κB, p62/SQSM1

## Abstract

In this study, we propose lactucopicrin (LCTP), a natural sesquiterpene lactone from Lactucavirosa, as a molecule able to control the growth of glioblastoma continuous cell line U87Mg. The IC50 of U87Mg against LCTP revealed a strong cytotoxic effect. Daily administration of LCTP showed a dose and time-dependent reduction of GBM cell growth and viability, also confirmed by inhibition of clonogenic potential and mobility of U87Mg cells. LCTP activated autophagy in U87Mg cells and decreased the phosphorylation of proliferative signals pAKT and pERK. LCTP also induced the cell cycle arrest in G2/M phase, confirmed by decrease of CDK2 protein and increase of p53 and p21. LCTP stimulated apoptosis as evidenced by reduction of procaspase 6 and the increase of the cleaved/full-length PARP ratio. The pre-treatment of U87Mg cells with ROS scavenger N-acetylcysteine (NAC), which reversed its cytotoxic effect, showed the involvement of LCTP in oxidative stress. Finally, LCTP strongly enhanced the sensitivity of U87Mg cells to canonical therapy Temozolomide (TMZ) and synergized with this drug. Altogether, the growth inhibition of U87Mg GBM cells induced by LCTP is the result of several synergic mechanisms, which makes LCTP a promising adjuvant therapy for this complex pathology.

## 1. Introduction

Glioblastoma (GBM) is one of the most lethal brain tumors in adults, with survival rates which have remained substantially unchanged for 30 years. Common histological features of GBM comprise marked mitotic activity, high angiogenesis, cellular heterogeneity, necrosis, and pronounced proliferative rates [1]. Furthermore, the presence of cancer stem cells, able to proliferate and generate glial neoplastic cells [2,3], contributes to the unfavorable prognosis of GBM patients, whose median survival is approximately 14 months [4]. Gross-total resection of tumor tissue, followed by adjuvant chemo- and radiotherapy, remains the standard of care [5]. Numerous efforts have been made to identify the molecular pathways and potential “druggable” targets involved in gliomagenesis [6].

Temozolomide (TMZ) is a first-line chemotherapy that has significantly improved the prognosis of GBM patients [7]. However, development of TMZ resistance in some GBM patients during the therapeutic program limits the treatment of this brain tumor. The main mechanism of GBM resistance involves O6-methylguanine-DNA methyltransferase (MGMT) enzyme repair. MGMT acts by removing the methyl group attached to the O6 position in guanine from DNA of cancer cells, favoring a resistant phenotype [8]. However, other mechanisms contribute significantly to TMZ resistance [9]. Several molecules have been developed to overcome TMZ resistance, such as O6-benzyl-guanine which inhibits MGMT, tyrosine kinase inhibitors which modulate the epidermal growth factor receptor (EGFR) commonly overexpressed in GBMs, and nutlin-3 which inhibits murine double minute 2 (Mdm2), that in turn leads to restoration of p53 activity.

Due to the failure of classical chemotherapies and targeted drugs, research efforts are focusing on natural compounds that can overcome BBB, inhibit tumor growth and are able to promote the activity involving multiple pathways [10]. In fact, since multiple pathways are associated with TMZ resistance in GBM [11], the treatment of GMB with a selective target drug that blocks the activation of a single pathway could result in a compensatory mechanism that leads to the restoration of signals, conferring a TMZ resistance [12]. Interestingly, multiple natural compounds have already shown antitumor and apoptotic effects in TMZ resistant GBM cell lines and also displayed synergistic affects with TMZ [10,13,14]. Sesquiterpene lactones are a large and different group of biologically active plant compounds with anti-inflammatory and anti-tumor activity. Sesquiterpene lactones are secondary metabolites of the Asteraceae family, which exhibits great structural diversity and a broad range of biological activities [15,16]. As a member of the sesquiterpene superfamily, they are composed by 15-carbon skeletons, consisting of colorless, crisp and dry lipophilic compounds made up of three isopropyl units [17,18]. The biological activities of sesquiterpene lactones are due to the presence of α-methylene-γ-lactone, which reacts with nucleophilic structure of multiple targets through Michael’s reaction, such as the thiol group of cysteinyl residues [17]. Therefore, they can exert their effects through the alkylation of transcription factors and various enzymes, interfering with several molecular pathways [19]. In the last few years, extensive research has confirmed the anticancer activity of different compounds within the sesquiterpene lactones and much effort has been undertaken to clarify the molecular mechanism and chemotherapeutic potential of some of these compounds in GMB treatment [20,21,22]. Very recently, Zhang et al. reported that lactucopicrin (or intybin), a secondary metabolite of lactucarium, derived from the plant *Lactuca virosa* (wild lettuce) and found in some related plants, such as Cichorium intybus, exhibits its anticancer activity in SKMEL-5 human skin cancer cells [23] and growth inhibition of Saos-2 osteosarcoma cells [24].

For a long time, lactucarium, a milky and bitter juice secreted by stem secretion of these plants, once dried, has been used as an opium substitute for its analgesic and sedative properties. Therefore, considering the ability of lactucopicrin to act on central nervous system, it can be speculated that this molecule can cross the BBB acting as multi-target molecule, potentially interfering with GMB cell growth. Therefore, we studied the effect of this natural substance to exploit the novel vulnerabilities of human glioblastoma cell line U87Mg.

## 2. Results

### 2.1. Dose-Response and Time-Course of LCTP Effects on Glioblastoma U87Mg Cells

**Figure 1:**
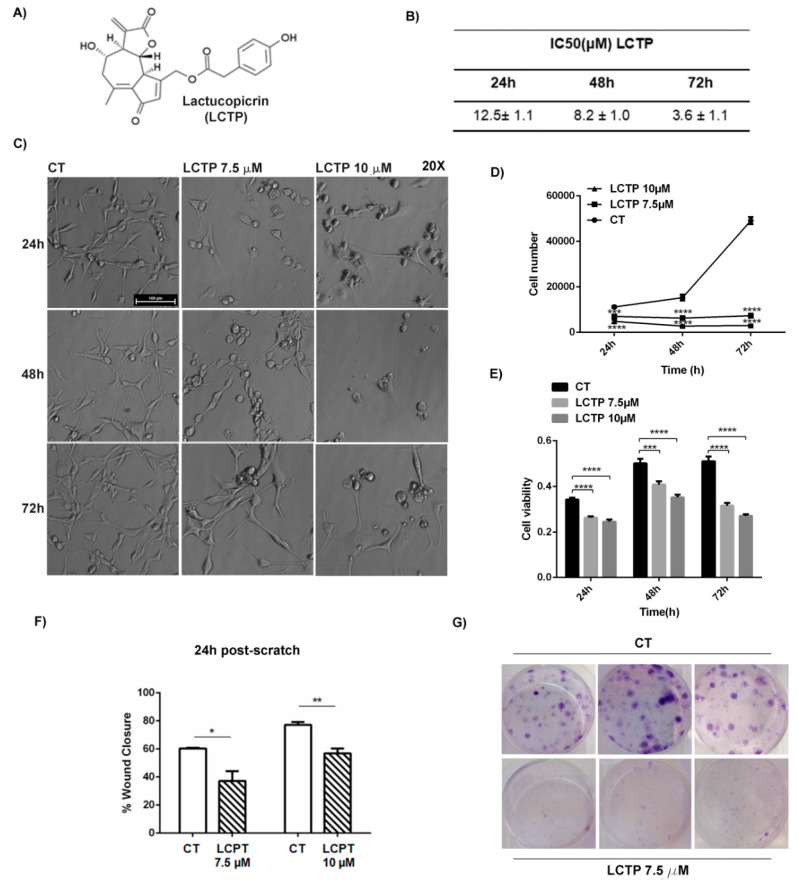
(**A**) Chemical structure of LCTP. (**B**) IC50-values of U87Mg cells after 24, 48 and 72 h of incubation with LCTP. (**C**) Morphological changes of U87Mg glioblastoma cells treated every 24 h with LCTP 7.5 and 10 µM for 24, 48 and 72 h. Magnification 20×. (**D**) Time and dose-dependent growth inhibition of LCT*P*-treated U87Mg cells. (**E**) Cytotoxic effect of various concentrations of LCTP (7.5 and 10 µM) at 24, 48 and 72 h pos*t*-treatment on U87Mg cell line. (**F**) Quantification of the cell-free area in wound healing assay at T0 and 24 h post-scratch in control and LCTP-treated U87Mg cells. (**G**) Colony-forming assay of U87Mg treated 24 h with LCTP 7.5. µM. For all the experiments, values are the means ± SEM of 3 individual determinations. Unpaired *t*-test, *p*-value < 0.05. According to GraphPad Prism, * *p*-value 0.01 to 0.05 (significant), ** *p*-value 0.001 to 0.01 (very significant), *** *p*-value 0.0001 to 0.001 (Extremely significant), **** *p*-value < 0.0001 (Extremely significant).

The cytotoxic effects of LCTP on glioblastoma were investigated using continuous glioblastoma cell line U87Mg and estimating the IC50 of LCTP at 24, 48, and 72 h. As it is evident in Figure 1B, the IC50 of LCTP against U87Mg cells decreased from 12.5 ± 1.1 µM (5.1 ± 0.5 µg/mL) at 24 h to 3.6 ± 1.1 µM (1.5 ± 0.5 µg/mL) at 72 h. Considering the strong cytotoxicity of this molecule, LCTP was daily administered at concentrations of 7.5 and 10 μM at various time intervals (24, 48 and 72 h). We found that LCTP exhibited a statistically significant time- and dose-dependent growth inhibition of U87Mg, as measured by direct cell count (Figure 1D), which resulted in a growth rate inhibition that increased from 60% and 82% at 48 h, respectively, with LCTP 7.5 and 10 μM to 85% and 94% of inhibition at 72 h of treatment. Cell viability assay and morphological changes were also reported in Figure 1C,E. According to the growth rate inhibition, LCTP inhibited the viability of U87Mg in a time and dose-dependent manner, with about 50% of reduction at 72 h of treatment with LCTP 10 μM. LCTP induced dramatic morphological changes of U87Mg, visible at microscopically observation as rounded-shaped with a loss of filaments and cell shrinkage (Figure 1C).

### 2.2. LCTP Interferes with Clonogenic Survival and Motility Capacity of GBM Cells

To further investigate the growth inhibition effects of LCTP, a colony assay of U87Mg treated with LCTP and control vehicle (DMSO) was performed. LCTP dramatically reduced the colony forming of U87Mg; at the lowest concentration used (7.5 µM), this natural molecule completely suppressed clonogenic growth (Figure 1G). LCTP also affected the cell motility of U87Mg, as it was demonstrated by the wound healing assay. The plot in Figure 1F showed that the percentage of wound closure significantly decreased with the increase of LCTP concentrations at 24 h post-scratch (Supplementary Figure S1).

### 2.3. Rapid Autophagy Response of U87Mg to LCTP Treatment Potentially Remodels the Cytoskeleton Proteins

**Figure 2:**
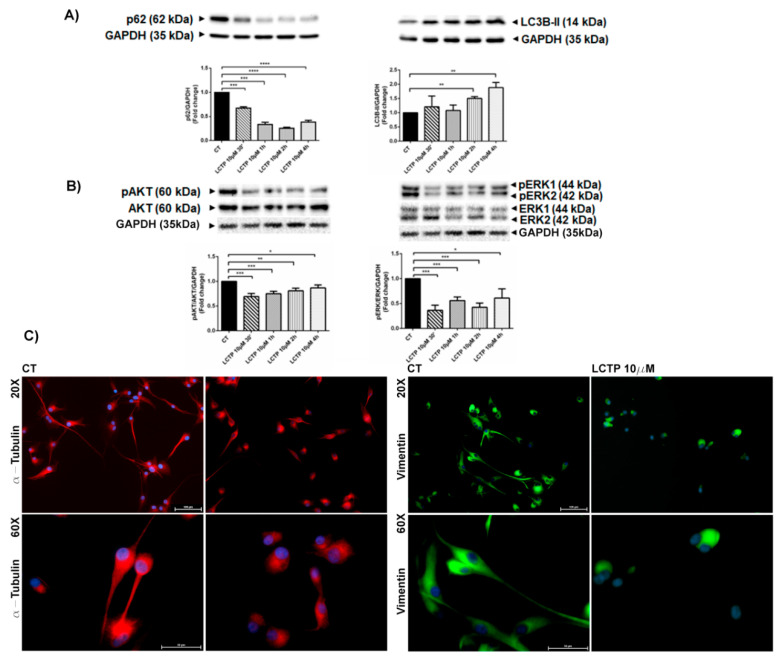
(**A**) Western blot analysis of autophagy-associated proteins p62 and LC3BII in short-termtreated-U87Mg cells with LCTP 10 µM. Densitometric analysis of protein levels represent the means ± SEM of 3 individual determinations. Data were normalized to GAPDH and are expressed as fold change over control-treated cells. * Unpaired *t*-test, *p*-value < 0.05. (**B**) Western blot analysis of proliferative signals pERK/ERK/GAPDH and pAKT/AKT/GAPDH in short-termtreated-U87Mg cells with LCTP 10 µM. Densitometric analysis of protein levels represent the means ± SEM of 3 individual determinations and are expressed as fold change over control-treated cells, normalized to GAPDH. * Unpaired *t*-test, *p*-value < 0.05. According to GraphPad Prism, * *p*-value 0.01 to 0.05 (significant), ** *p*-value 0.001 to 0.01 (very significant), *** *p*-value 0.0001 to 0.001 (Extremely significant), **** *p*-value < 0.0001 (Extremely significant). (**C**) Immunofluorescence analysis of cytoskeleton proteins α-tubulin and Vimentin in U87Mgcells treated with LCTP 10 µM and vehicle control (DMSO 0.08%) for 20 min. Magnification 20× and 60×.

The molecular mechanisms underlying LCT*P*-induced cytotoxicity were investigated exposing U87Mg cells to short-term treatment with LCTP 10 µM. In the Western blot analysis, in Figure 2A, the strong reduction of autophagic substrate p62/SQSM1 appears evident, already being visible after 30 min of induction followed by the increased of cleaved LC3BII, clearly indicating the activation of autophagy in GBM cells treated with LCTP, with respect to control cells. Autophagy activation may be sustained by a deep dephosphorylation of ^Ser473^pAKT and pERK1/2 proteins in a LCT*P*- short-term treatment of U87Mg cells (Figure 2B). Interestingly, LCTP induced a strong cytoskeleton rearrangement in U87Mg cells, as it can be appreciated by immunofluorescence staining for intermediated filament Vimentin and the α-tubulin subunit of microtubules (Figure 2C).

### 2.4. Cell Cycle Arrest in G2/M Phase and Apoptosis Induction by LCTP in U87Mg Cells

**Figure 3:**
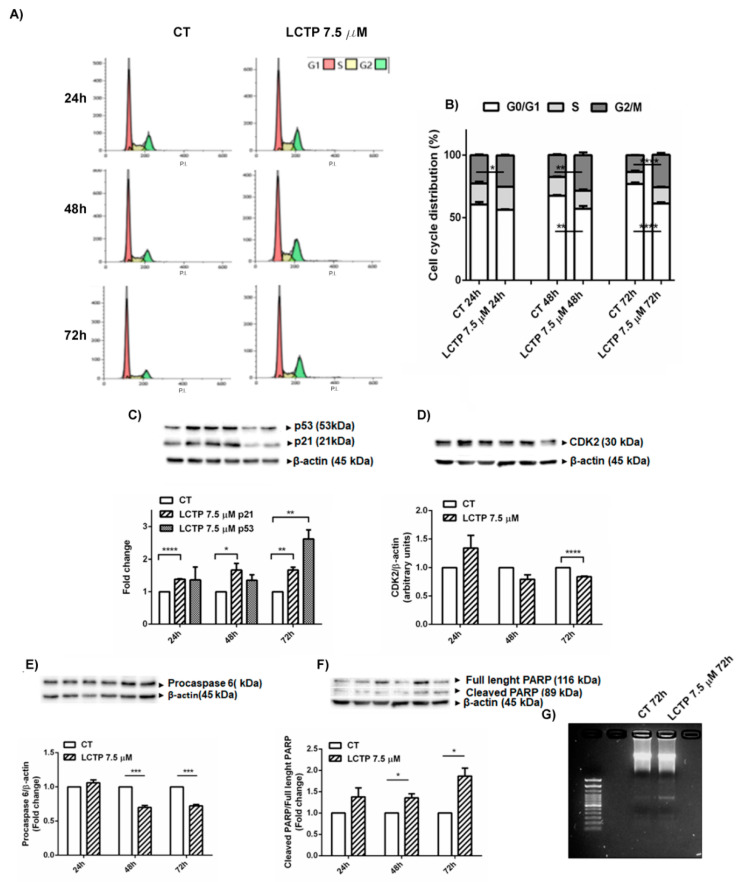
(**A**) Representative flow cytometry analysis of cell cycle arrest in G2/M and (**B**) plot of cell cycle distribution of propidium iodide (PI) staining in U87Mg, vehicle (left panel) and LCTP (7.5 µM) treated cells (right panel) for 24, 48 and 72 h. Values are the means ± SEM of 3 individual determinations. * Unpaired *t*-test, *p*-value < 0.05. (**C**) Western blot analysis of p53 and p21 in long-term LCTP--treated U87Mg cells. Data were normalized toβ-actin and are expressed as fold change over control-treated cells of 3 individual determinations. * Unpaired *t*-test, *p*-value < 0.05. (**D**) Western blot analysis of CDK2: the figure shows CDK2 analysis of U87Mg cells treated with LCTP 7.5 µM at different time (24, 48, and 72 h). Data were normalized to β-actin and are expressed as fold change over control-treated cells of 3 individual determinations. * Unpaired *t*-test, *p*-value < 0.05. (**E**) Western blot and densitometric analysis of procaspase 6 of LCT*P*-treated U87Mg cells at different time (24, 48 and 72 h). Values are the means ± SEM of 3 individual determinations and protein expression levels, normalized to β-actin, are expressed as fold change over control-treated cells. * Unpaired *t*-test, *p*-value < 0.05. (**F**) Western blot analysis of PARP in LCT*P*-treated U87Mg cells at different time (24, 48 and 72 h). Values as ratio of cleaved PARP to full-length PARP represent the means ± SEM of 3 individual determinations and are expressed as fold change over control-treated cells. * Unpaired *t*-test, *p*-value < 0.05. According to GraphPad Prism, * *p*-value 0.01 to 0.05 (significant), ** *p*-value 0.001 to 0.01 (very significant), *** *p*-value 0.0001 to 0.001 (Extremely significant), **** *p*-value < 0.0001 (Extremely significant). (**G**) DNA laddering of long-term treatment of U87Mg cells with LCTP 7.5 with respect to untreated cells.

To clarify the mechanism that regulates GBM cell growth inhibition, the long-term effects of LCTP on cell cycle distribution were investigated by flow cytometry. The analysis revealed that the cell cycle arrest in G2/M phase in LCTP-induced U87Mg cells was already visible at 24 h post-treatment (Figure 3A). The cell cycle distribution showed that the arrest in the G2/M phase—and concomitant decrease of cell percentage at the G0/G1 phase—is time-dependent (Figure 3B). In order to clarify the molecular mechanism underlying the growth inhibition of the U87Mg cells, the expression levels of key proteins which regulate the cell cycle were determined by Western blot analysis. As shown in Figure 3C, the long-term treatment of U87Mg cells with LCTP 7.5 µM induced the activation of CDK inhibitor p21, which was already up-regulated after 24 h of treatment. The tumor suppressor p53 was strongly activated at 72 h post-treatment in accordance, with the pronounced accumulation of cell population in the G2/M phase at the same time point (Figure 3B,C). The up-regulation of p21 and p53, after 72 h, is correlated to CDK2 inhibition in LCTP-treated cells, confirming the cytostatic activity of LCTP in U87Mg cells (Figure 3D). In the same conditions, to further investigate whether the anti-proliferative effect of LCTP was accompanied by the induction of cell death, Western blot analysis of apoptotic proteins was performed. In U87Mg-treated cells at 48 and 72 h post-treatment, procaspase 6 decreased, while cleaved/full-length ratio of Poly(ADP)ribose polymerase (PARP) significantly increased, clearly indicating the activation of programmed cell death by LCTP (Figure 3E,F). Loss of repair activity of cleaved PARP was also confirmed by the appearance of DNA fragments, as shown on 2% agarose gel electrophoresis at 72 h of LCTP-treatment (Figure 3G).

### 2.5. Involvement of Oxidative Stress in LCTP-Mediated Cytotoxicity in U87Mg Cells

**Figure 4:**
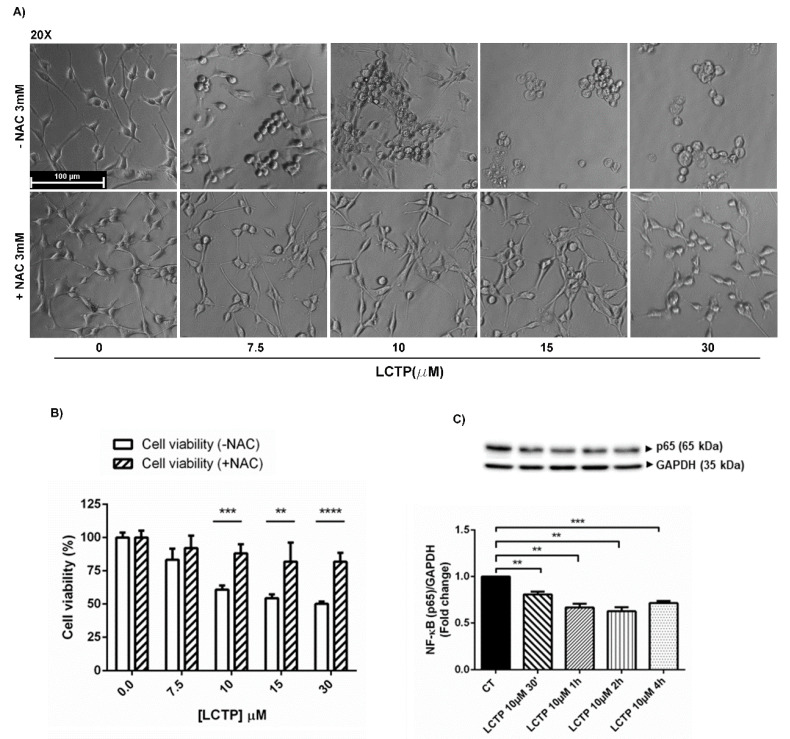
(**A**) Effect of pre-treatment with ROS scavenger NAC 3 mM for 4 h on morphological changes and (**B**) cell viability (%) induced by different concentrations of LCTP (7.5, 10, 15 and 30 µM) on U87Mg cells at 24 h from treatment. Magnification 20×. (**C**) Western blot analysis of NF-κB p65 subunit of shor*t*-term treated U87Mg cells with LCTP 7.5 µM (30 min, 1 h, 2 h and 4 h). Data were normalized to GAPDH and are expressed as fold change over control-treated cells of 3 individual determinations. According to GraphPad Prism, ** *p*-value 0.001 to 0.01 (very significant), *** *p*-value 0.0001 to 0.001 (Extremely significant), **** *p*-value < 0.0001 (Extremely significant).

The involvement of oxidative stress in LCT*P*-induced cytotoxicity is due to the presence of highly reactive α-methylene-γ-lactone group. This group is able to react with nucleophilic structure of multiple targets through Michael’s reaction, such as the thiol group of cysteinyl residues [17]. The oxidative stress LCT*P*-induced cytotoxicity was demonstrated by pre-incubating U87Mg with N-acetylcysteine. Pre-treatment with ROS scavenger NAC reverted the LCTP effects on U87Mg cell viability and preserved cell morphology (Figure 4A,B). These results suggest that LCTP induces a redox imbalance that potentially mediates its anti-proliferative activity. ROS can influence tumor cell malignancy via the redox-regulated transcription factor NF-κB [25]. Therefore, Western blot analysis of short-term LCT*P*-treated GBM cells was performed to assess the expression levels of NF-κB p65 subunit. The down-regulation of NF-κB p65 expression upon LCTP treatment was already visible after 30 min of incubation with the drug and persisted throughout all of the time tested (1 h, 2 h, and 4 h) (Figure 4C). The expression of NF-κb p65 at long-term treatment was also tested (Supplementary Figure S2).

### 2.6. LCTP Enhances the Sensitivity of U87Mg to Canonical Therapy Temozolomide

**Figure 5:**
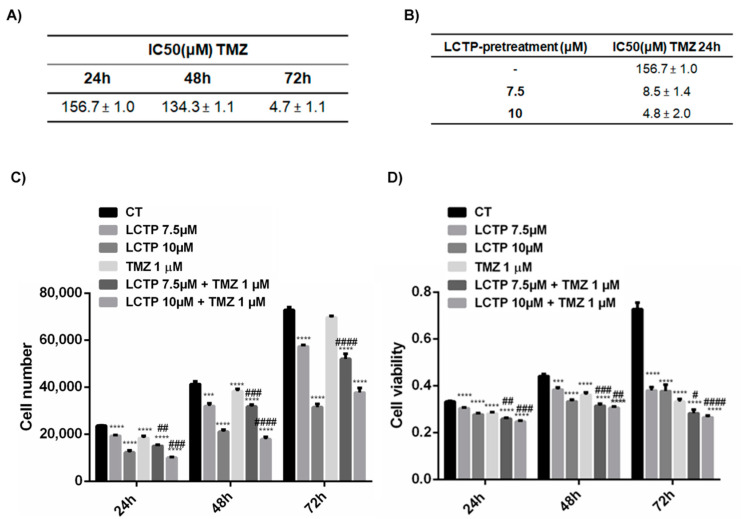
(**A**) IC50-values of U87Mg cells after 24, 48 and 72 h of incubation with TMZ. (**B**) IC50-values of TMZ at 24 h of U87Mg cells pre-treated with LCTP 7.5 and 10 µM for 24 h. (**C**) Synergistic effect of LCTP and conventional chemotherapy TMZ ongrowth of U87Mg cells. Values are the means ± SEM of 3 individual determinations. # *p*-value compared to cells treated with TMZ alone. (**D**) Synergistic effect of LCTP and conventional chemotherapy TMZ oncell viability of U87Mg cells. Values are the means ± SEM of 3 individual determinations. # *p*-value < 0.05 compared to cells treated with TMZ alone. According to GraphPad Prism, # *p*-value 0.01 to 0.05 (significant), ## *p*-value 0.001 to 0.01 (very significant), *** or ### *p*-value 0.0001 to 0.001 (Extremely significant), **** or #### *p*-value < 0.0001 (Extremely significant).

To assess whether the effects of LCTP can influence the response to TMZ, U87Mg cells were pre-treated with LCTP 7.5 µM and 10 µM for 24 h and IC50 for TMZ at 24 h, which was determined to be compared with the standard IC50. As shown in Figure 5A,B, the IC50 of TMZ at 24 h was about 18 and 32 times higher compared to IC50 for TMZ at 24 h when U87Mg cells were pre-treated with LCTP (7.5 µM and 10 µM). It is worth noting that the lower IC50 of U87Mg to LCTP if compared with TMZ at 24 and 48 h, indicating a strong effect of LCTP in a short-term period, which could explain the rapid activation of autophagic pathway. At 72 h, the IC50 for TMZ and LCTP of U87Mg cells became comparable (Figure 1B and Figure 5A).

### 2.7. Synergist Effect of LCTP and TMZ Affects the Cell Growth and Viability

In order to propose LCTP as an adjuvant therapy for GBM, in combination with conventional chemotherapy TMZ, U87Mg cells were treated every 24 h with LCTP 7.5 µM and 10 µM in combination with TMZ 1 µM for 24, 48. and 72 h. The effects of the combined therapy were assessed on cell growth and viability. Concomitant treatment with the two drugs significantly increased the effect of TMZ alone, inhibiting the replicative potential of U87Mg cells already from 24 h post-treatment (Figure 5C,D).

## 3. Discussion

The current oncology protocol for glioblastoma patients provides, after surgery, treatment with chemotherapeutic agents, such as TMZ, associated with radiotherapy (Stupp protocol) [5]. Over the past 10 years, however, therapeutic agents have not significantly increased the median survival rate of patients with glioblastoma. The 5-year survival rate for patients with the same disease, after treatment including surgical resection, radiotherapy, and chemotherapy, remains less than 9.8% [26].

Therapeutic approaches used against glioblastoma are associated with the development of resistance and with important side effects, which can very often represent a real obstacle for the patient in completing chemotherapy, and this can lead to therapeutic failure. For this reason, the current research is moving towards using natural substances as adjuvants to a traditional therapy that can, on the one hand, have an antitumor action (synergistic with temozolomide) and, on the other, soothe the side effects. A large number of clinical studies have demonstrated the benefits derived from the use of herbal medicines in combination with conventional therapies on the survival, immune modulation, and quality of life of cancer patients [27]. Here, we examined the anti-proliferative effects of a natural compound, Lactucopicrin, commonly found in Lactuca virosa, on human glioblastoma cells U87Mg. The effects of LCTP on the proliferation of GBM continuous cell line U87Mg were evaluated by setting up growth curves. To study the effect of LCTP on the growth rate of human glioma cells U87Mg, we applied the drug at chosen concentration (7.5 and 10 µM) to the growing medium once daily for three days, starting one day after plating. These applications had already reduced the growth in culture after only 24 h post-treatment. A time- and dose-dependent growth rate inhibition was observed in U87Mg upon LCTP treatment. This trend was also confirmed by results obtained for cell viability assay. In accordance with results from cell count and MTT assay, the growth inhibitory effect of LCTP on U87Mg cell was also confirmed by clonogenic assay. In fact, at the lower concentration chosen (7.5 µM), LCTP completely suppressed the potential clonogenicity of U87Mg cells. Migration is a peculiar aspect which favors the aggressiveness and poor prognosis glioblastoma [28]; in this regard, our results evidence a dose-dependent, anti-migratory effect of LCTP on U87Mg cells, since the ability of cells to close the scratch decreased with LCTP concentrations.

The half-maximal inhibitory concentration of LCTP against U87Mg was determined at different time points, revealing a strong cytotoxic effect of LCTP. The IC50 for LCTP in the micromolar range is in line with the previously reported data in human SKMEL-5 [23]. Interestingly, compared with conventional chemotherapy TMZ, the IC50 of LCTP at 24 and 48 h resulted in being more than 10 times lower, becoming comparable only at 72 h of treatment.

In order to evaluate the potential synergism of TMZ with LCTP, the preliminary ability of this natural molecule to arrest the cell proliferation of U87Mg cells was investigated by comparing the IC50-values for TMZ before and after treatment with LCTP. LCTP strongly increased the sensitivity of U87Mg to TMZ. Finally, to test the synergism with TMZ (canonical drug for GBM) and to propose LCTP as adjuvant therapy, the GBM cell growth and viability were analyzed upon combined treatment which demonstrated a synergistic effect. We considered that it would be interesting to investigate the mechanisms involved in inhibiting cell growth after LCTP administration. Since members of the sesquiterpene lactone family have been reported to induce S and G2/M cell cycle arrest and cell death inducing apoptosis through PARP cleavage [20,23,29], the ability of LCTP to induce the cell cycle arrest in GBM U87Mg cells was evaluated by flow cytometry analysis. The results demonstrated a block of the cell cycle in the G2/M phase, especially 72 h after treatment with LCTP. The cell cycle arrest was supported by the increased expression of the CDK inhibitor p21 and oncosuppressor p53, while CDK2 protein decreased. The cell cycle blockage is not to be considered as the only mechanism that determines the growth inhibition of U87Mg cells after treatment with LCTP. In this order, the molecular activation of the apoptotic and autophagic responses, as well as the key pathways of proliferation U87Mg continuous cell line, were assessed by Western blot analysis of GBM cell extracts after treatments. Long-term exposure to LCTP 7.5 µM revealed an increase cleaved/full-length PARP ratio, confirming an involvement of the apoptotic pathway which is already visible by procaspase 6 decrease at 48 and 72 h of induction with LCTP.

The pro-apoptotic and cytostatic effects of LCTP may be the results of a rapid activation of autophagic pathway. The autophagy adaptor p62/SQSM1 has been reported to act as an oncogene in glioma and has been proposed as a novel therapeutic target for this pathology [30]. Moreover, a decreased expression of p62/SQSM1 induced a significant decrease of ERK phosphorylation [31]. According to previous literature, in our study, Western blot analysis revealed a strong and rapid reduction of p62/SQSM1 accompanied by the increase of cleaved LC3BII and a remarkable dephosphorylation of ERK1/2 kinases in LCTP--treated cells. The presence of α-methylene-γ-lactone promotes the nucleophilic attack of sesquiterpene lactones to multiple targets through Michael’s reaction, such as the redox regulator NF-κB [17]. The constitutive activation of this transcription factor has been shown to stimulate the growth and survival of GBM [32]. Several natural sesquiterpene lactones act as NF-kB inhibitors [33], such as parthenolide and artemisinin, which enhance the sensitivity of cancer cells to chemotherapy [34,35]. In this regard, the effect of LCTP on the NF-kB p65 subunit was investigated by Western blot analysis, that revealed a significant reduction of p65 expression levels already appreciable at 30 min from induction. According to Jing et al., the autophagic degradation of p62/SQSM1 can lead the inhibition of NF-kB pathway and thus inhibit the proliferation of U87Mg glioblastoma cells [36]. The high nucleophilic reactivity of LCTP against cysteine-reactive electrophiles and the involvement of oxidative stress in LCTP-induced cytotoxicity was investigated pre-incubating U87Mg cells with NAC, a cysteine precursor acting as ROS scavenger. NAC completely reversed the cytotoxic effects of LCTP and protected cells from morphological changes.

AKT represents a central point of the RTK/PTEN/PI3K pathway. High levels of AKT and phospho-AKT have been detected in the majority of GBM tumor samples and cell lines, where it supports uncontrolled glioma cells growth, apoptotic blockage, and tumor invasion, thus representing an attractive pathway for GBM targeting therapy [37]. Western blot analysis displayed, in cells treated with LCTP for 30 min,1 h, 2 h, and 4 h, a decrease in AKT phosphorylation, implying a strong reduction of the proliferation signaling pathway.

Finally, our study evidenced that treatment of U87Mg cells with LCTP induced—already in the first 30 min of treatment—a profound reshape of the glioblastoma cells’ cytoskeleton, leading to the development of autophagic structures [38,39]. Immunofluorescence analysis revealed a profound change in α-tubulin distribution, that appeared clearly distributed in the cytoskeletal structures of the microtubules in the control glioblastoma cells, while α-tubulin is very concentrated in the cytoplasm after treatment with LCTP. On this side, LCTP treatment also remodeled the intermediate filament Vimentin, which appeared normally distributed in the cytoplasmic long stress fibers in control cells while accumulating near the nucleolemma, conferring a cell rounded-shaped morphology in LCTP-treated cells.

Concluding, the LCTP can be considered an excellent adjuvant substance for the treatment of glioblastoma, as it is involved in a series of mechanisms that control the growth of glioblastoma cells: autophagy, cell cycle arrest, and oxidative stress. Furthermore, the reduction of transcription factor NF-κB p65—a critical regulator of immune and inflammatory responses—showed a potential role of LCTP into reducing chronic inflammation. This natural substance—which potentially can pass through the BBB—used in combination with the canonical chemotherapy Temodal, opens the door to a multimodal therapy which could prove to be the most effective weapon against complex diseases, such as glioblastoma.

## 4. Materials and Methods

### 4.1. Cell Culture

The continuous human glioblastoma cell line U87Mgwas obtained from Sigma Aldrich Collection (LGCPromochem, Teddington, UK). U87Mg cells were growth in Dulbecco’s Modified Eagle’s Medium supplemented with 10% fetal bovine serum, 2 mmol/L-glutamine, 100IU/mL penicillin, 100 μg streptomycin at 37 °C, 5% CO_2_, and 95% of humidity. For in vitro treatment, the pure molecule LCTP (Extrasynthase, Genay, France) and Temozolomide (Sigma Aldrich, St. Louis, MO, USA) were used.

### 4.2. Estimation of Half—Maximal Inhibitory Concentration (IC50) of LCTP and TMZ in U87Mg Cells

To estimate the IC50-values of LCTP and TMZ at 24, 48, and 72 h, U87Mg cells were plated in 96-well plates at density of 5 × 10^3^ cells/well. The IC50-values for the selected drugs were determined by using, respectively, the concentrations of 7.5, 10, 15, and 30 μM for LCTP and 10, 50, 100, 150, and 200 μM for TMZ. The IC50-values for LCTP and TMZ in U87Mg cells were calculated using GraphPad Prism (GraphPad Software Inc., San Diego, CA, USA).

### 4.3. Proliferation Assay

The in vitro response of U87Mg cells to LCTP were evaluated by plating human GBM cell line in 48-well plates at 1 × 10^4^ cells/well in DMEM supplemented with 10% FBS, incubating them at 37 °C in an atmosphere containing 5% CO_2_. The following day, the cells were treated every 24 h with LCTP at concentrations of 7.5 μM and 10 μM for 24, 48, and 72 h. DMSO 0.08% was used as vehicle control. At the selected time point, cell count was performed using a Burker chamber.

### 4.4. Cell Viability Assay

U87Mg cells were seeded at density of 5 × 10^3^ cells in 96-well plates. The cultures were treated every 24 h with concentrations of LCTP 7.5 μM and 10 μM for 24, 48, and 72 h, followed by MTT (3-(4,5-dimethylthiazol-2-yl)-2,5-diphenyltetrazolium bromide) (Sigma–Aldrich) assay. DMSO 0.08% was used as vehicle control. Briefly, 5 mg/mL MTT was added in 100 µL of cultured cells in DMEM medium. Formazan crystals were dissolved in isopropanol/HCl 0.4% and the absorbance measured at 570 nm with a plate reading spectrophotometer.

LCTP-induced oxidative stress in U87Mg cells was investigated pre-treating starved cells with N-acetylcysteine (NAC) 3 mM for 4 h at 37 °C in DMEM with FBS 10%. After 4 h, the medium was replaced and GBM cells treated with different concentrations of LCTP (7.5, 10, 15, and 30 µM) for 24 h. DMSO 0.2% was used as vehicle control.

### 4.5. Microscopic Observation of Cell Morphology

U87Mg cells were seeded in 96-well plates and incubated with LCTP 7.5 µM every 24 h in DMEM with FBS 10% for 24, 48, and 72 h in the presence or absence of N-acetylcysteine 3 mM. After treatment, morphological changes of U87Mg were observed and imaged by a phase contrast microscope (Evos, Life technologies, Carlsbad, CA, USA).

### 4.6. Combined Treatment of TMZ and LCTP in Human GBM Cell Line

The in vitro response of U87Mg cells to combined LCTPand TMZ treatment was assessed seeding GBM cells in 48-well plates (10,000 cells/well) and treating the cells every 24 h with TMZ 1 μM alone or in combination with LCTP 7.5 μM and 10 μM for 24, 48, and 72 h. At the end of each treatment, cells were counted using a Burker chamber. The combined cytotoxic effects of LCTP and TMZ were evaluated by MTT assay as above described: U87Mg cells were plated at density of 5 × 10^3^ cells in 96-well plates and treated with the same combination of drugs (LCTP 7.5 μM and 10 μM in combination with TMZ 1 μM) for 24, 48, and 72 h.

### 4.7. Estimation of IC50 of TMZ after Pre-Treatment with LCTP

To test the ability of LCTP to enhance the sensitivity of U87Mg cells to TMZ, cells were plated in 96-well plates at density of 5 × 10^3^ cells/well and pre-treated for 24 h with LCTP 7.5 μM and 10 μM, before the estimation of IC50-values for TMZ. Cells were incubated with concentrations of TMZ 10, 50, 100, 150, and 200 μM and IC50-values for TMZ at 24 h determined by using GraphPad Prism (GraphPad Software Inc., San Diego, CA, USA).

### 4.8. Wound Healing Assay

To evaluate cell motility, GBM cells were seeded into 6-well culture plates. When the cells reached 90% confluence, a scratch was gently made through the cell monolayer by sterile 100 μL pipette tips, and the detached cells were washed away. The cell migration was observed and imaged under an Evos FL microscope (Life Technologies) for each condition (LCTP 7.5 μM and 10 μM) and timepoint (T0 and T24 h).

### 4.9. Clonogenic Assay

The clonogenic assay was performed seeding 10^3^ U87Mg cells/well in triplicate in 6-well plates for 48 h. Cells were treated with LCTP 7.5 μM and control vehicle (0.08% DMSO) for 24 h and the medium was replaced every 3 days for 14 days. The colonies were fixed with 4% paraformaldehyde solution for 5 min, washed with PBS, and stained with crystal violet 0.05% for 30 min.

### 4.10. Western Blot Analysis of LCTP-Treated U87Mg Cells

Protein extraction from LCTP-treated U87MG cells was performed with Triton X-100 lysis buffer (Tris-HCL 10 mM, EDTA 1 mM, NaCl 150 mM, Triton X-100 1%, NaF 1 mM, Na_4_P_2_O_7_ 1 mM, Na_3_VO_4_ 1 mM, and protease inhibitors 1×). Protein lysates (15 μg) were resolved on a 12.5% SDS-PAGE transferred to PVDF membranes by electroblotting. The membranes were incubated for 1 h at room temperature in 5% not-fat dry milk or bovine serum albumin (BSA) diluted in 1× Tris-buffered saline containing Tween-20 (TBST) and then incubated overnight at 4 °C with primary selected antibodies. For protein normalization, each membrane was then incubated with mouse monoclonal anti–β-actin (1:10,000, Santa Cruz Biotechnology, Santa Cruz, CA, USA) or anti-GAPDH (1:1000, Santa Cruz Biotechnology). The membranes were incubated with the specific HRP-conjugated secondary antibodies (Calbiochem). The protein bands were detected by chemiluminescence using ECL Western blotting (Amersham) and the digital signals were quantified by densitometric analysis using Image Lab Software (Bio-Rad Laboratories, Hercules, CA, USA). For cell cycle proteins CDK2, p21, and p53 analysis, the cells were plated at a density of 5 × 10^5^ cells in60 mm plates in DMEM without FBS for 48 h. After re-adding 10% FBS, inductions were performed every 24 h with LCTP7.5 μM and the cells collected after 24, 48, and 72 h of treatment. Anti-CDK2, anti-p21 antibody (1:1000, Cell Signaling) and anti-p53 (1:1000, Roche) were used.

Changes in the phosphorylation status of ERK1/2 and AKT, in the expression level of autophagy-associated proteins p62 and LC3B and NF-κB-p65 subunit were evaluated by treating U87Mg cells with LCTP 10 μM for 30 min, 1 h, 2 h, and 4 h. The membranes were incubated overnight with anti-pERK1/2 and anti-pAKT antibody (1:1000, Cell Signaling) in 2.5% bovine serum albumin (BSA) in TBST. The membranes were stripped to be re-probed with non-phosphorylated forms of anti-ERK1/2 and anti-AKT (1:1000, Cell Signaling). For autophagic proteins, anti-p62 (1:1000, Cell Signaling) in 2.5% milk in TBST and LC3B (1:1000, Cell Signaling) in BSA 2.5% in TBST were used. For NF-κB –p65 (1:1000, Santa Cruz) subunit in milk 2.5% in TBST were used.

### 4.11. Western Blot of Apoptosis-Associated Proteins

LCTP-induced apoptosis in U87Mg cells was assessed by Western blot analysis of caspases 6 and Poly (ADP-ribose) polimesare (PARP). The samples were prepared as previously reported for cell cycle proteins analysis and the PVDF membranes were incubated with caspase 6 (Cell Signaling, 1:1000), and PARP (Cell Signaling 1.100) in 2.5% milk in TBST overnight at 4 °C. The incubation with secondary antibodies and detection of proteins were performed as above described.

### 4.12. Immunofluorescence

U87Mg cells (5 × 10^3^) were plated in 8-well chamber slides in DMEM with 2% serum for 48 h. Cells were treated with LCTP 10 µM in DMEM with 10% FBS for 20 min and the morphological change induced by LCTP treatment assessed by immunofluorescence staining for cytoskeleton proteins, α-tubulin, and Vimentin. In detail, at the end of treatment, U87Mg cells were fixed in 4% formalin (Diapath) for 20 min and permeabilized with 0.1% Triton (Invitrogen, Carlsbad, CA, USA) for 30 min. After blocking with 10% specific serum, the cells were incubated with anti-Vimentin (prediluted; Roche diagnostic) and anti-α-tubulin (1:200, Abcam, Cambridge, MA, USA) overnight at 4 °C. After washing with 0.025% PBS-Tween-20, cells were incubated with secondary antibody anti-mouse fluorescein (1:100; Vector, Stuttgart, Germany) in 2% serum for 1 h at room temperature. The slides were counterstained with DAPI Mounting Medium (Vectashield) for nuclei detection and analyzed with a fluorescence microscope at 20× and 40× magnification.

### 4.13. Cell Cycle Analysis by Flow Cytometry

U87MG human GBM cells were plated (25 × 10^4^) in DMEM with 2% serum for 48 h and treated with LCTP 7.5 µM every 24 h in DMEM with FBS 10% for 24, 48, and 72 h. After treatment, cells were trypsinized, washed in sample buffer (glucose 0.1% in HBSS), fixed in 70% ethanol, and stored at 4 °C overnight until the day of analysis. Before analysis, Propidium iodide (50 μg/mL) was added for 30 min at room temperature. Flow cytometry analysis of the cell cycle was performed with a Gallios instrument (Beckman Coulter, Brea, CA, USA).

### 4.14. DNA Laddering

U87Mg cells were plated (4.5 × 10^4^) in DMEM with 2% FBS for 48 h and induction performed every 24 h with LCTP: 7.5 µM and DMSO 0.08% as vehicle control for 72 h. At the end of treatment, the cells were collected, and the pellets were washed with phosphate buffer saline. DNA was extracted with QIAamp DNA Blood Mini kit (Qiagen, Hilden, Germany) and the DNA fragments were separated by 2% agarose gel electrophoresis and visualized by an SYBR Safe DNA Gel Stain (Invitrogen).

### 4.15. Statistical Analysis

Experiments were performed in triplicate and data were expressed as mean ± SEM. Statistical significance was determined by Student’s *t*-test, considering a *p*-value of < 0.05 statistically significant.

## Supplementary Materials

The following are available online. Figure S1(A) Representative images of wound healing assay of U87Mg cells treated with vehicle (DMSO 0.08%) and LCTP 7.5 and 10 µM at T0 and 24 h post-scratch (T24). Figure S2 Western blot analysis of NF-κB p65 subunit of long-term treated U87Mg cells with LCTP 7.5 µM for 24, 48, and 72 h. Data were normalized to β-actin and are expressed as fold change over control-treated cells of 3 individual determinations. * Unpaired *t*-test, *p*-value < 0.05.
