# Supplementary figures and images for: Implication of Lactucopicrin in Autophagy, Cell Cycle Arrest and Oxidative Stress to Inhibit U87Mg Glioblastoma Cell Growth
Source: Molecules. 2020 Dec 10;25(24):5843. doi: 10.3390/molecules25245843 (PMC7764785; doi:10.3390/molecules25245843)

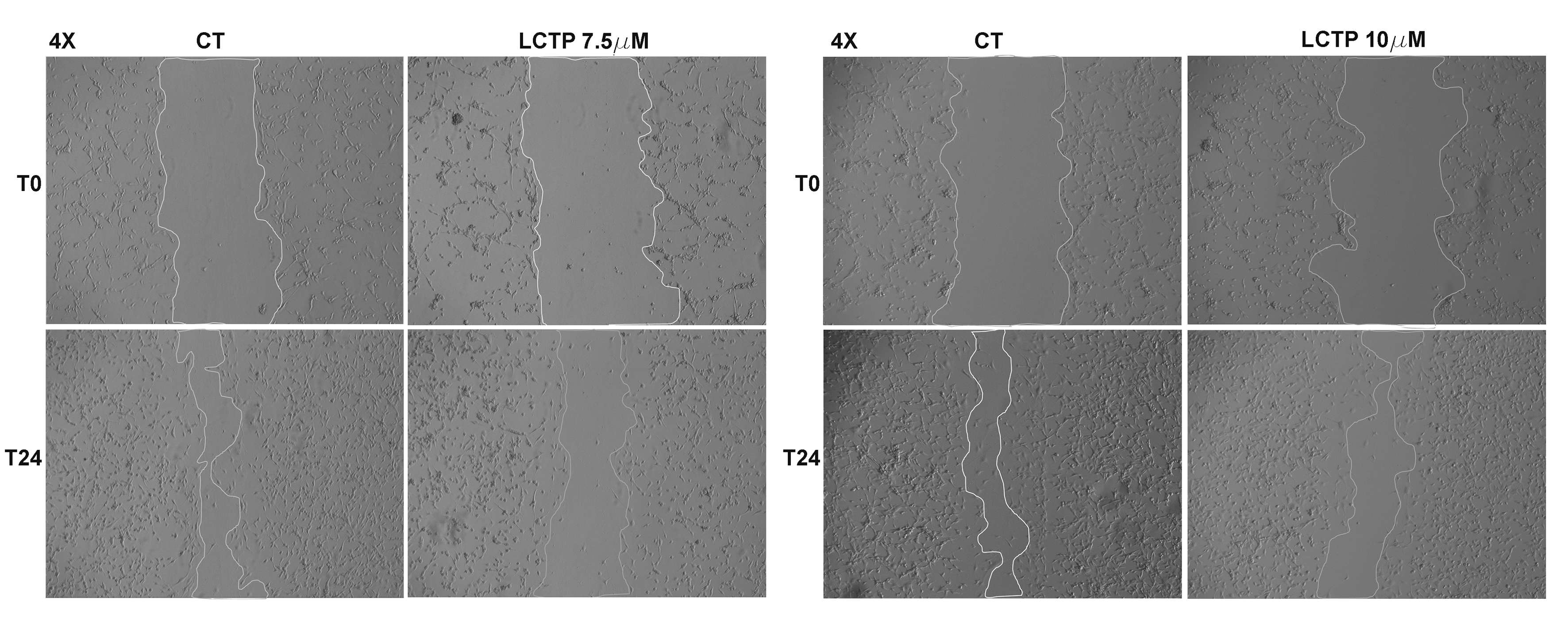

Supplement: Supplementary file 1 [file molecules-25-05843-s001.zip › Figura 1S LCTP.tif]

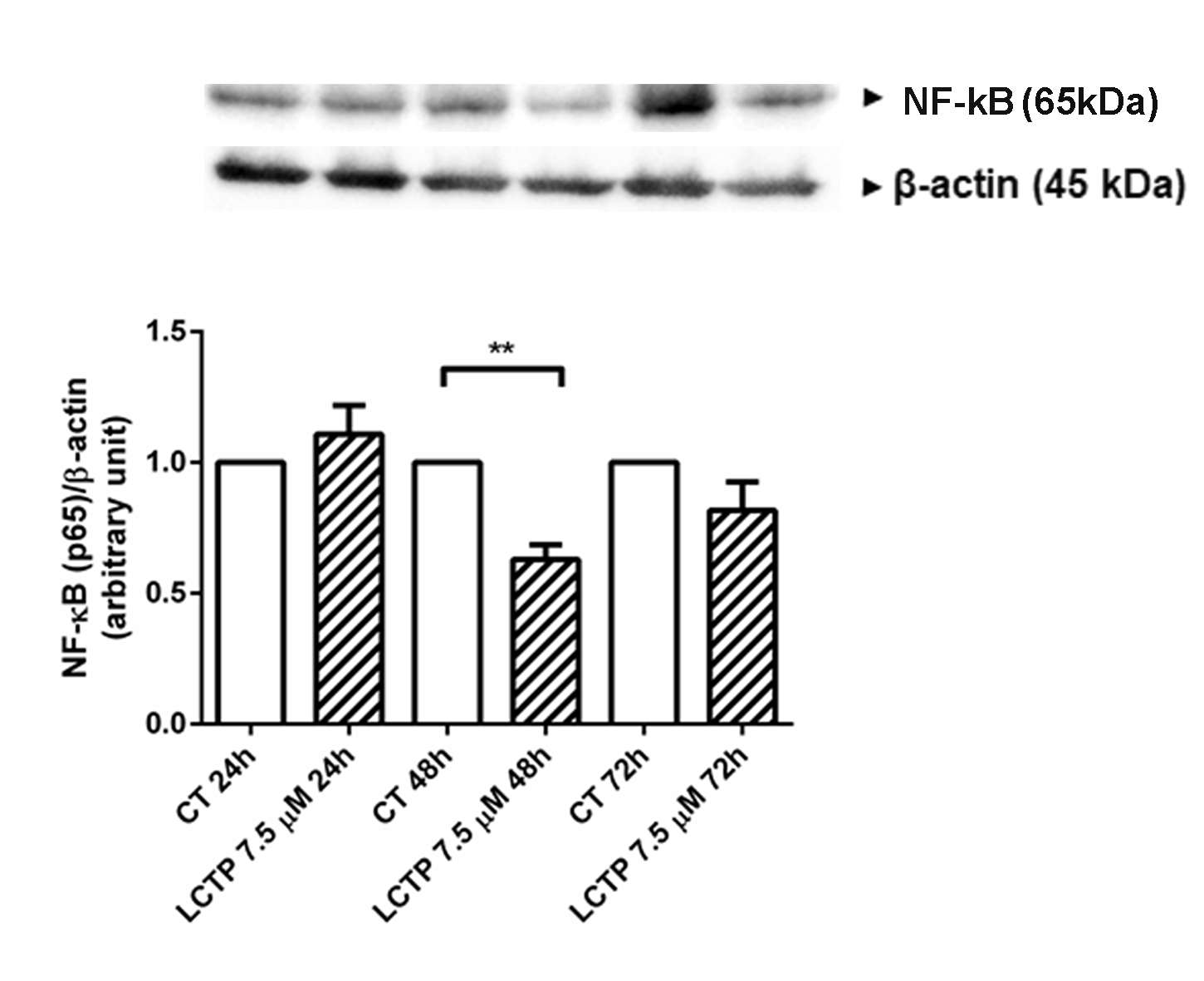

Supplement: Supplementary file 1 [file molecules-25-05843-s001.zip › Figure 2S.tif]
